# Supplementary material for: A mathematical model of local and global attention in natural scene viewing
Source: PLoS Comput Biol. 2020 Dec 14;16(12):e1007880. doi: 10.1371/journal.pcbi.1007880 (PMC7769622; doi:10.1371/journal.pcbi.1007880)
Supplement: S1 Text — In this appendix you can find the technical details of the Gibbs sampler described in the Methods section. It includes a derivation of the full likelihood, details regarding the augmentation schemes and the derivation of the conditional distributions. (PDF) [file pcbi.1007880.s001.pdf]

S1 Appendix: Parameter Inference of the Exploration Exploitation Model in Natural Scene Viewing

Noa Malem-Shinitski[1], Manfred Opper[2], Sebastian Reich[1], Lisa Schwetlick[3], Stefan A. Seelig[3‡], Ralf Engbert[3‡],

1 Institute of Mathematics, University of Potsdam, Potsdam, Germany
2 Department of Artificial Intelligence, Technische Universität Berlin, Berlin, Germany
3 Department of Psychology, University of Potsdam, Potsdam, Germany

* malem@uni-potsdam.de

1 Likelihood augmentation

In the Methods section we defined the likelihood function of the model and the prior distributions over the model parameters. Here, we present the details of the inference process for the estimation of the posterior distribution.

The full likelihood function of the model is

$$p(Z,\Gamma|\Theta) = p(z_1)p(z_2)\prod_{t=3}^{T} p_{\text{exploit}}\left(z_t|z_{t-1}\right)^{\gamma_t}\ p_{\text{explore}}\left(z_t|z_{t-1}\right)^{1-\gamma_t}\ p\left(\gamma_t\right) \tag{1}$$

with

$$\begin{aligned} p_{\text{exploit}}\left(z_t|z_{t-1}\right) &= \frac{n\left(z_t; z_{t-1}, \epsilon\right)}{\sum_{z'} n\left(z'; z_{t-1}, \epsilon\right)} \\ p_{\text{explore}}\left(z_t|z_{t-1}\right) &= \frac{\max\left(s\left(z_t\right) n\left(z_t; z_{t-1}, \xi\right) - n\left(z_t; z_{t-1}, \epsilon\right), 0\right)}{\sum_{z'} \max\left(s\left(z'\right) n\left(z'; z_{t-1}, \xi\right) - n\left(z'; z_{t-1}, \epsilon\right), 0\right)} \end{aligned}$$

where

$$\begin{aligned} \gamma_t \sim \text{Bern}\left(\rho_t\right) =&\ \text{Bern}\left(\sigma\left(f\left(s\right)\right)\right) \\ =& \left(\frac{1}{1+\exp\left(-f\left(s\right)\right)}\right)^{\gamma_t} \left(\frac{\exp\left(-f(s)\right)}{1+\exp\left(-f\left(s\right)\right)}\right)^{1-\gamma_t} \\ =& \frac{1}{1+\exp\left(-f\left(s\right)\right)} \exp\left(-f\left(s\right)\right)^{1-\gamma_t} = \frac{2\exp\left(\left(\gamma_t - \frac{1}{2}\right)\frac{f(s)}{2}\right)}{\cosh\left(\frac{f(s)}{2}\right)} \end{aligned}$$

and

$$f\left(s\right) = b\left(\frac{s_{t-1}}{s_{t-2}} - s^o\right). \tag{2}$$

We choose the following prior distributions

$$\begin{aligned} \epsilon_{x/y} &\sim \text{IG}\left(\alpha_{\epsilon_{x/y}}, \beta_{\epsilon_{x/y}}\right) \\ \xi_{x/y} &\sim \text{IG}\left(\alpha_{\xi_{x/y}}, \beta_{\xi_{x/y}}\right) \\ b &\sim \mathcal{N}\left(\mu_b, \sigma_b\right) \\ s^o &\sim \mathcal{N}\left(\mu_{s^o}, \sigma_{s^o}\right). \end{aligned}$$

We wish to derive a Gibbs sampler to estimate the posterior distribution. To do so we need to derive the conditional distribution for each of the model parameters. This is not possible in the current form of the posterior distribution and we use an augmentation technique. The augmentation process described below results in an exponential quadratic form in b and s^o which allows for simple sampling from their respective conditional distributions.

In the process of data augmentation one introduces a set of auxiliary variables to make the model conditionally conjugated [1]. In this work we augment the model with Polyá-gamma variables W. We add one w_t for each data point z_t. The Polyá-Gamma variables are defined in the following way [2, 3].

The random variable $w \sim PG(1,0)$ is defined by its moment generating function:

$$\mathbb{E}\left(\exp(-tw)\right) = \cosh^{-1}\left(\sqrt{\frac{t}{2}}\right). \tag{3}$$

We set $t = \frac{f(s)^2}{2}$ and apply Equation 3 to Equation 1 which results in the following simplified posterior for the augmented model

$$p\left(\Theta, W, \Gamma | Z\right) \propto p\left(z_1\right) p\left(z_2\right) \prod_{t=3}^{T} p_{\text{exploit}}\left(z_t | z_{t-1}\right)^{\gamma_t} p_{\text{explore}}\left(z_t | z_{t-1}\right)^{1-\gamma_t} \times \exp\left(-\frac{f\left(s\right)^2}{2} w_t + \left(\gamma_t - \frac{1}{2}\right) f\left(s\right)\right) p\left(w; 1, 0\right) p\left(\Theta\right) \tag{4}$$

with:

$$\Theta = \{b, s^o, \epsilon, \xi\} \tag{5}$$

$$p\left(\Theta\right) = p(b)p(s^o)p(\epsilon)p\left(\xi\right). \tag{6}$$

We use the posterior distribution above in the Gibbs sampler and we next describe how to sample from the full conditionals of W, Γ and the model parameters Θ.

2 The Conditionals

2.1 w_t

It is not immediately clear how to sample from the conditional distribution for w_t. A helpful step is to identify the factors which depend on w_t in Equation 4 as part of the exponential tilted distribution of $w \sim PG(1,0)$. $w \sim PG(1,c)$ is the exponential tilting of $w \sim PG(1,0)$, and using the moment generating function introduced in Equation 3:

$$p(w; 1, c) = \frac{\exp(-\frac{c^2}{2}w)p(w|1,0)}{\mathbb{E}\left(\exp\left(-\frac{c^2}{2}w\right)\right)} = \cosh\left(\frac{c}{2}\right)\exp\big(-\frac{c^2 w}{2}\big)p\left(w; 1, 0\right). \tag{7}$$

Setting $c = f\left(s\right)$ for each w_t we conclude that to sample from the conditional distribution for w_t we need to sample from $w \sim PG\left(1, f\left(s\right)\right)$. For the details of the sampling process see [4]. In this work we used the Python implementation of the sampler described by Windel et al. called "pypolyagamma" and is available under https://github.com/slinderman/pypolyagamma .

2.2 γ_t

To sample γ_t we calculate the Bayes Factor and sample from $\text{Bern}(\gamma_t; \rho_t)$ with:

$$\begin{aligned}\rho_t &= \frac{\sigma\left(f\left(s\right)\right)\ p\left(z_t|\gamma_t = 1\right)}{\sigma\left(f\left(s\right)\right)\ p\left(z_t|\gamma_t = 1\right) + \left(1 - \sigma\left(f\left(s\right)\right)\right)\ p\left(z_t|\gamma_t = 0\right)} \\ &= \frac{\sigma\left(f\left(s\right)\right)}{\sigma\left(f\left(s\right)\right) + \text{BF}\left(1 - \sigma\left(f\left(s\right)\right)\right)}\end{aligned} \tag{8}$$

and BF defined as:

$$\text{BF} = \frac{p\left(z_t|\gamma_t = 0\right)}{p\left(z_t|\gamma_t = 1\right)} = \frac{p_{\text{explore}}\left(z_t|z_{t-1}\right)}{p_{\text{exploit}}\left(z_t|z_{t-1}\right)} \tag{9}$$

2.3 b and s^o

Taking into account the definition of $f\left(s\right)$ we see that the parameters b and s^o appear in linear and quadratic forms in the arguments of the exponents in Equation (4). Given the Gaussian prior distributions we chose, the conditional distributions of these parameters are also Gaussian

$$p(b|Z, W, \Gamma, s^o, \epsilon, \xi) = n\left(b; m_b, s_b\right) \tag{10}$$

with mean and variance

$$m_b = \sum_{t=1}^{T}\left(\gamma_t - \frac{1}{2}\right)\left(\frac{s_{t-1}}{s_{t-2}} - s^o\right) + \frac{\mu_b}{\sigma_b}s_b \tag{11}$$

$$s_b = \frac{\sigma_b}{1 + \sigma_b \sum_{t=1}^{T} w_t \left(\frac{s_{t-1}}{s_{t-2}} - s^o\right)^2}. \tag{12}$$

Similarly

$$p(s^o|Z, W, \Gamma, b, \epsilon, \xi) = n\left(s^o; m_{s^o}, s_{s^o}\right) \tag{13}$$

with mean and variance

$$m_{s^o} = \left(\sum_{t=1}^{T}\left(b^2 w_t \frac{s_{t-1}}{s_{t-2}} - b\left(\gamma_t - \frac{1}{2}\right)\right) + \frac{\mu_{s^o}}{\sigma_{s^o}}\right)s_{s^o} \tag{14}$$

$$s_{s^o} = \frac{\sigma_{s^o}}{1 + \sigma_{s^o} b^2 \sum_{t=1}^{T} w_t}. \tag{15}$$

2.4 ϵ and ξ

Due to the complex form of p_{explore}, we do not have a closed form for the conditional distributions of ϵ and ξ from which we can sample. As an estimate we draw a sample using the Hamiltonian Monte Carlo (HMC) algorithm. The HMC algorithm requires an energy function and its derivative. In our case we use the negative log of the model likelihood as the energy function. Rather than calculating the derivative of the log likelihood analytically we use automatic differentiation ([5]). Specifically we use the Python Autograd package. We further tuned the step size and number of steps parameters of the leapfrog algorithm to achieve an acceptance rate of 100%. Specifically we used a step size of 0.03 for ϵ and 0.5 for ξ with eight leapfrog iterations for both.

References

1. Tanner MA, Wong WH. The calculation of posterior distributions by data augmentation. Journal of the American Statistical Asociation. 1987;82:528–540.

2. Polson NG, Scott JG, Windle J. Bayesian Inference for Logistic Models Using Pólya–Gamma Latent Variables. Journal of the American Statistical Association. 2013;108(504):1339–1349.

3. Choi HM, Hobert JP. The Pólya-Gamma Gibbs sampler for Bayesian logistic regression is uniformly ergodic. Electronic Journal of Statistics. 2013;7:2054–2064.

4. Windle J, Polason NG, Scott JG. Sampling Pólya-gamma random variates: alternate and approximate techniques. Preprint arXiv:1405.0506. 2014.

5. Griewank A. On automatic differentiation. Mathematical Programming: Recent Developments and Applications. 1989;6(6):83–107.
